# Radiotherapy and immune response: the systemic effects of a local treatment

**DOI:** 10.6061/clinics/2018/e557s

**Published:** 2018-11-27

**Authors:** Heloisa de Andrade Carvalho, Rosangela Correa Villar

**Affiliations:** I Departamento de Radiologia e Oncologia, Divisao de Radioterapia, Faculdade de Medicina FMUSP, Universidade de Sao Paulo, Sao Paulo, SP, BR; II Servico de Radioterapia, Centro de Oncologia, Hospital Sirio-Libanes, Sao Paulo, SP, BR; III Servico de Radioterapia, Centro Infantil Boldrini, Campinas, SP, BR

**Keywords:** Radiotherapy, Immunotherapy, Abscopal Effect, Tumor Cell Death, Cancer Treatment

## Abstract

Technological developments have allowed improvements in radiotherapy delivery, with higher precision and better sparing of normal tissue. For many years, it has been well known that ionizing radiation has not only local action but also systemic effects by triggering many molecular signaling pathways. There is still a lack of knowledge of this issue. This review focuses on the current literature about the effects of ionizing radiation on the immune system, either suppressing or stimulating the host reactions against the tumor, and the factors that interact with these responses, such as the radiation dose and dose / fraction effects in the tumor microenvironment and vasculature. In addition, some implications of these effects in cancer treatment, mainly in combined strategies, are addressed from the perspective of their interactions with the more advanced technology currently available, such as heavy ion therapy and nanotechnology.

## INTRODUCTION

Radiotherapy is one of the cornerstone treatment modalities for cancer. It is also the most commonly used cancer treatment strategy, with approximately 60% of patients with solid tumors receiving curative or palliative irradiation as part of their treatment 1.

High precision techniques are currently available, making treatment delivery more safe and effective, while sparing adjacent normal tissue 2.

Radiation-induced cell death is modulated by several factors related to the molecular physicochemical effects that induce cellular stress. Depending on the dose and irradiation schedules used, cellular senescence or death will occur as a result of the disequilibrium caused by the stress 3.

The tumor microenvironment, especially the degree of oxygenation, has also long been known to influence the response to radiation. This has broadened the studies on radiobiology that currently include the effects of radiation on tumor vasculature, fibroblasts and immune cells 4.

There is growing interest in research on immunotherapies based on the critical role of the immune system in cancer development and dissemination 5. The anticancer immune response may also be activated by ionizing radiation, and a combination of different treatment strategies is promising in this field 3,6.

The objective of this manuscript was to provide a brief update about the role of radiotherapy in cell death and the activation of the antitumor immune system from the perspective of clinical applications in oncologic treatment.

### Radiation-induced cell death

The effect of radiation on biological material is due to the absorption of energy from X-rays or gamma rays, or from charged particles within the atoms that may be ionized or excited, initiating a chain of actions that lead to a final biological effect 6. Within tumor cells, DNA is the main target of radiation. The so-called lethal effect is caused by a direct action of radiation causing DNA damage. Radiation may also act indirectly by interacting with other atoms and molecules in the cell, mainly water, called “radiolysis of water”, to produce free radicals that are able to trigger chemical reactions with different targets. This indirect action produces sublethal or potentially lethal damage and may lead to cell death 7. The different cellular mechanisms triggered after exposure to ionizing radiation cell death may be expressed in different ways. Galluzzi et al. 7, in 2007, proposed three main modalities of cell death that may be ordered according to morphological, biochemical and enzymatic processes as follows: apoptosis – type I, autophagic cell death – type II, and necrosis or necroptosis – type III.

### Apoptosis

Apoptosis also called programed cell death is characterized by classical morphological alterations such as cellular shrinkage, chromatin condensation (pyknosis), nuclear fragmentation (karyorhexis), plasma membrane blebbing, and the formation of apoptotic bodies that are engulfed by neighboring phagocytes very quickly without inducing inflammatory processes 8. Apoptosis is the most important form of radiation-induced cell death, and the following two distinct signaling pathways may be involved in its activation: the intrinsic and the extrinsic pathways, driven by intracellular signals such as DNA damage and metabolic alterations and extracellular signals such as death ligands, respectively 3. A cascade of caspases is activated in both pathways. These cascades act in both apoptosis mechanisms and other cellular processes that are independent of cell death modalities. 9.

### Autophagic cell death

Autophagic cell death is a concept that should be differentiated from autophagy. Autophagy is a regulatory mechanism that acts to maintain cellular homeostasis and to protect genomic integrity through a lysosomal pathway. The lysosomes prevent the accumulation of aggregated and misfolded proteins as well as the action of damaged organelles and helps in the maintenance of homeostasis 10. Many anticancer therapies, including chemotherapy and radiotherapy, may induce autophagy in tumor cells. However, depending on the cellular context at the time and the type of treatment, the treatment response may inhibit or favor tumor progression in an autophagy-mediated manner by autophagy-related (ATG) proteins 11,12. In addition, at least some portion of radiation-induced apoptosis is dependent on the autophagic process in some tested cell lines, and some reports demonstrated that cell death after irradiation could be decreased by the inhibition of autophagy 13.

Autophagic cell death, on the other hand, is defined as a distinct process that occurs after the induction of cell autophagy 14. This may be explained by the finding that inhibitors of autophagy function and/or genetic inactivation of autophagic modulators may block autophagic cell death 15. However, in some situations, it can occur in another way. In a recent publication, the protein ATG5, for example, was implicated in the induction of autophagic cell death after irradiation 13, a process rendered distinct from the induction of autophagy by the inhibition of the mTOR and kinase AKT pathway 12.

Thus, during cancer treatment, autophagy can contribute to the regulation of the fate of cancer cells 3.

### Necrosis or necroptosis

Necrosis is another form of cell death characterized by organelle swelling, plasma membrane rupture, and cell lysis with loss of intracellular content 16. This form of cell death leads to the release of intracellular components and causes an intense inflammatory response not observed in other forms.

The term “necroptosis” was proposed to indicate a regulated (not accidental) process of necrosis. Necroptosis may be defined as a type of cell death that can be avoided by inhibiting kinase RIP1, a cell death mediator (either through genetic or pharmacological methods) 17. These mediators may represent a way to discriminate programmed from uncontrolled forms of necrosis 17. Therefore, strong evidence suggests that necrosis is not an uncontrolled process but may be regulated by a number of signal transduction pathways and catabolic mechanisms, as has been described in other forms of cell death 17,18.

Radiation-induced death is particular to each cell tissue and tumor, and the prevalence of the death of some types of cells over other cells can be changed by the dose. Radiation in low doses generally eliminates cancer cells through apoptosis, while high doses can lead to necrosis in other cancer cells, such as osteosarcoma cells 19.

### Atypical radiation-induced cell death modalities

Atypical cell death modalities are defined as those that do not or that only partially exhibit the morphological features, biochemical alterations and enzymatic activities described. They are less studied and are called mitotic catastrophe and senescence 3.

Mitotic catastrophe is also a response that can be induced after irradiation in cells. It occurs in cells with impaired p53. These cells have problems in the repair process because they are not able to properly activate the cell cycle checkpoints to initiate cell cycle arrest and DNA repair. The DNA lesion promoted by irradiation, deficient cell cycle checkpoints and repair, and hyperamplification of centrosomes contribute to mitotic catastrophe. Apoptosis and mitotic catastrophe are among the most frequent forms of cell death due to radiation 20.

The cells have an aberrant nuclear morphology, often resulting in aneuploid and polyploid cell descendants that almost always die 21,22. Cancer cells containing unrepaired, defective DNA enter prematurely into mitosis and experience mitotic catastrophe 22. Anti-proliferative actions can be utilized to stop proliferation of cells with defective mitosis. When this protective mechanism is impaired, unrestricted growth of defective cells, such as tumor cells, may occur, facilitating tumor development 21,23.

Senescence is defined as a widespread and permanent cell cycle arrest after the proliferative capacity of the cells is exhausted. This process is considered an antitumor barrier that prevents cancer cell proliferation 3. However, senescent cells are still viable and metabolically active 17, 21. They can secrete many pro-inflammatory cytokines, chemokines, growth factors, and proteases that collectively are known as the senescence-associated secretory phenotype (SASP). These can have positive effects or not on cellular proliferation. Therefore, while senescence represents a cell-autonomous tumor suppressor mechanism, radiation-induced senescence could impact the surrounding cells and favor tumor survival and growth 3.

### Nontargeted effects of radiation

Allied to these primary effects of radiation on cell death, mostly promoted by DNA lesions, other secondary effects can also be observed. The main effect seems to be due to the activation of the immune system via the induction of immunogenic cell death by ionizing radiation. Radiation is able to modify tumor phenotypes and the tumor microenvironment as well. Anti-tumor responses may also be mediated by these nontargeted effects in a specific and systemic manner and have the ability to target both relapsing tumor cells and distant metastases 21,24-26.

### Cancer and immune system

Tumors develop in a microenvironment composed of cells of the immune system. The immune system can modulate either tumor suppression or progression. Cancer immunoediting comprises the dual host-protective and tumor-promoting actions of immunity 27,28. Cancer immunoediting is a dynamic process composed of the following three phases: elimination (i.e., cancer immunosurveillance), equilibrium, and escape. This process is also known as the three “Es” of cancer immunoediting 29.

In the first phase, elimination, cells and molecules of the innate (natural killer cells – NK, and macrophages) and adaptive (dendritic cells – DC, CD8+ and CD4+ T lymphocytes) immunity, which comprise the cancer immunosurveillance network, interact with the highly immunogenic tumor cells that are present at the beginning of the carcinogenic process. These immune cells may eradicate the growing tumor and protect the host from tumor formation. However, due to genetic instability, cells that are more resistant to the immune attack may emerge but remain under the constant control of the immune system, which is the equilibrium phase. Maintained chronically or immunologically in this immune “editor” environment, they may produce new populations of tumor variants. These variants may occasionally escape the immune system by different mechanisms and become clinically detectable in the escape phase. At that point, not only do the tumor cells present fewer antigens but they also promote the recruitment of immunosuppressive cells that inhibit the local effector cells. Among them, T regulator (Treg) lymphocytes, myeloid-derived suppressor cells (MDSCs), the secretion of indoleamine 2,3-dioxygenase (IDO) and cytokines such as interleukine-10 (IL-10) are the most important in the establishment of a highly immunosuppressive environment. In this way, the tumor escapes from immunosurveillance and becomes clinically detectable 29,30.

### Ionizing radiation and the immune system

The effects of ionizing radiation are seen not only in the tumor cells but also in the tumor microenvironment. In general, lymphocytes (T cells, B cells and NK) are among the most radiosensitive cells, followed by monocytes, macrophages and antigen-presenting cells (APCs), specifically dendritic cells (DC), which have a higher radioresistance 31-33. Ionizing radiation also has an effect on the vascular endothelium, with an increase in the production of molecules involved in cellular adhesion, which facilitates the recruitment of antitumor T cells against the corresponding sites 34.

After irradiation, dead and stressed cells release a variety of substances that gives ionizing radiation either immunosuppressive or immune stimulating properties. There is still a lack of information on the role and functionality of immune cells after irradiation. Nevertheless, a number of experimental studies have clarified some aspects of the immune response after exposure to radiation.

Radiation induces distinct tumor cell death forms and, consequently, the release of pro-inflammatory cytokines, chemokines, tumor antigens, and other danger signals. Through this mechanism, radiation may enhance tumor immunogenicity. Radiation may promote a large amount of tumoral neoantigens that are then presented to the T lymphocytes. Therefore, radiation carries the potential to initiate the adaptive and innate immune responses, resulting in systemic antitumorigenic effects inside and outside of the irradiation field 35.

The observed regression of metastases or tumors outside the irradiation field is called the abscopal effect, and its relationship with immune events has been known since 1969^36^. The abscopal effect is partially mediated by the immune system, and T cells are the cells elected to mediate distant tumor immune inhibition induced by radiation 37. More recently, this radiation-induced cell death that causes an immune reaction has also been called “immunogenic death” 38.

After cell death, pro-inflammatory mediators are released. They are called damage-associated molecular patterns (DAMPs). Among them, we have reactive oxygen (ROS) and nitrogen species, cytotoxic cytokines tumor growth factor β-1 (TGβ-1), tumor necrosis factor-alpha (TNF-α), a number of interleukins, heat shock proteins (HSPs), high mobility group box 1 molecules (HMGB1), and nucleotides or uric acid are capable of activating the innate or adaptive immune system 39. These DAMPs are recognized by the Toll-like receptors (TLR) expressed on the surface of the DCs and are responsible for their activation and maturation 40. Tumor infiltrating DCs are associated with either good or poor prognosis in different cancer types. Although they seem to be quite radioresistant, radiation may cause a functional impairment of DCs, possibly leading to a change in the DC-mediated balance between T-cell activation and tolerance 41.

Adenosine-5-triphosphate (ATP) is another inflammatory molecule associated with immunological cell death. ATP binds to the DC receptors and can stimulate the release of interleukin-1β (IL-1β), which can promote T cell priming. Moreover, ATP released from tumor cells also modulates the immunosuppressive properties of myeloid-derived suppressor cells (MDSCs) and contributes to tumor growth 42.

The MDSCs together with other cells such as tumor-infiltrating macrophages or tumor-associated macrophages (TAMs) can contribute to tumor growth and inhibit antitumor immunity 3. This paradoxical immunosuppressive effect of radiation is mainly due to the inactivation of NK lymphocytes, with the recruitment of MDSCs and Treg lymphocytes, secretion of TGF-β and the modification of the macrophage phenotype 43.

**Figure 1:**
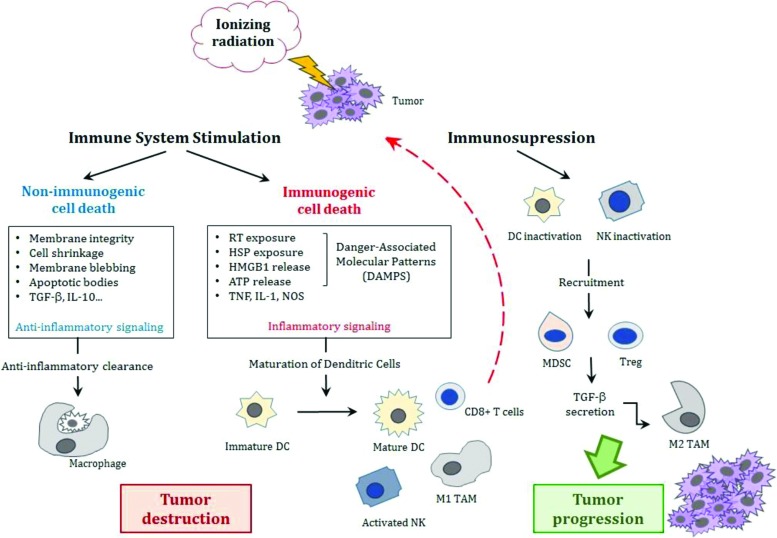
The effects of ionizing radiation effects on the immune system. Either stimulation or suppression of the immune system occurs. Stressed cells may simply undergo anti-inflammatory clearance resulting in non-immunogenic cell death or trigger inflammatory signaling that will release damage-associated molecular patterns (DAMPs) with the activation of dendritic cells that initiate cytotoxic T-cell responses against tumor cells. On the other hand, inactivation of these cells (DCs and cytotoxic T-cells) with the recruitment of MDSCs and T regulator lymphocytes and the secretion of TGF-β leads to the modification of the macrophage phenotype from a pro-inflammatory type M1 to an immunosuppressive type M2 that may allow tumor growth and progression. [Adapted from Derer et al., 2015 44 and Bockel et al., 2017 93]. Abbreviations: TGF-β, tumor growth factor-β; IL, interleukin; RT, radiotherapy; HSP, heat shock proteins; HMGB1, high mobility group box 1 molecules; ATP, adenosine-5-triphosphate; TNF, tumor necrosis factor; NOS, nitrogen reactive species; DC, dentritic cells; NK, natural killer; MDSC, myeloid derived suppressor cells; Treg, T regulator lymphocyte; TGF-β, tumor growth factor-β; TAM, tumor-associated macrophages.

A summary of these paradoxical immune responses after irradiation is represented in Figure 1. Understanding the role of these cells in the anticancer immune response is important for the development of anticancer therapies. Cell death causes an intense inflammatory response due to the release of intracellular components.

### Effects of ionizing radiation on the tumor microenvironment (TME)

Currently, we recognize that ionizing radiation effects not only affect cancer cell and cancer cell death but also the complex biological interactions between tumors and stroma in which they grow, known as the tumor microenvironment (TME). It is becoming increasingly evident that responses that are triggered within TME may be critical in determining the success or failure of therapy 44.

### Tumor vasculature

Endothelial cells and the tumor vasculature are possibly the best studied components involved in the effect of radiation on the TME. Radiation induces endothelial cell dysfunction, which is characterized by increased permeability, detachment from the underlying basement membrane and apoptosis 45. This effect is observed especially with high single-fraction doses (8–16 Gy) that induce endothelial cell apoptosis by the upregulation of acid sphingomyelinase (ASMase) 45. Thus, endothelial cell dysfunction and apoptosis caused by radiation contribute to postirradiation inflammation and fibrosis. Within vessels, irradiation also generates a pro-thrombotic state characterized by platelet aggregation, microthrombus formation and increased adhesion of inflammatory cells to endothelial cells with subsequent diapedesis into the perivascular space 46.

Structurally, irradiation of the vasculature can cause the destruction of blood vessels. The main structure affected is microvasculature 45,47, and the effect is dose-dependent. In addition to the total dose and fraction size, other important points involved are the tumor type, location and stage. All these factors are related to the effect of radiation on the vasculature 45,48. Tumor hypoxia, which could be a factor for tumor radioresistance, can be potentiated by the vascular damage caused by radiation. However, it generates an increased production of cytokines/chemokines that induces immune cell recruitment and triggers immune responses 45.

### Effects of radiation dose and fractionation on immune activation

The effects of radiation on the tumor microenvironment and immune system may be modified by the radiation dose and the dose delivery methods used. The model of tumor control by radiation is mainly based on cell damage due to direct and indirect effects. This model was developed with the conventional standard dose fractionation of 2 Gy per fraction 49. However, the effects of radiotherapy on the TME and on the antitumoral immune response described in preclinical studies has led to the concept of an existing immunogenic cell death (ICD) and immune-mediated tumor rejection 50,51.

To date, a variety of hypotheses about the specific impact of different dose/fractionation regimens on the anti-tumoral response are under investigation. In preclinical studies, the use of hypofractionated high doses rather than high single dose schedules showed the best results regarding the pro-immunogenic effect of radiation. 50,52,53.

In addition, larger doses should have more pro-immunogenic effects regarding the induction of ICD in *in vitro* studies 54. However, the relationship of the immune response with dose and fractionation may be more complex, as suggested by *in vivo* studies 55,56. The ability of radiotherapy to trigger anti-tumor T cells is influenced by the pre-existing TME and by the effects of radiotherapy on the TME 45. Currently, a consensus about the optimal dose schedule to stimulate the immune system has not yet been achieved with preclinical data that have been published. The use of single large doses (e.g., 30 Gy), as well as the 2 Gy standard dose, or even the classic hypofractionated doses of 6 or 8 Gy delivered daily on consecutive days have all been described as being effective 55-57.

Therefore, the radiation and immunotherapy partnership is completely dependent on the radiation dose and fraction involved.

### Association of radiotherapy with immunotherapies

Many immunotherapies were tested in combination with radiotherapy in preclinical studies and are now under investigation in the clinical setting 52. Among them, several immunological manipulation treatments have been used, including immune checkpoint blockade, adaptive T cell transfer, cytokine therapy, dendritic cell and peptide vaccines, and monoclonal antibodies.

The induction of anti-tumor immunity seems to be regulated step by step by positive and negative signals. Immunotherapies that have been currently approved and those in development act at one or multiple steps of this process. Radiotherapy seems to potentially accentuate each step, including the uptake of tumor antigens by dendritic cells and their activation, as well as migration of the activated effector T cells back to the tumor. Therefore, radiotherapy enhances and complements the action of many different immunotherapy agents, and its synergistic use is the goal of many exploratory studies 52,57-59.

### Radiotherapy techniques and perspectives

With respect to the tolerance dose of normal tissue and side effects, conventional regimens of radiation treatments deliver an effective dose of 40 to 70 Gy to achieve tumor control, depending on the tumor type, in daily doses of 1.8 to 2 Gy/day, delivered over several weeks. The advances in radiotherapy machines and planning systems have allowed the delivery of highly accurate treatments with high precision. Currently, it is possible to deliver higher doses per fraction with better sparing of the adjacent normal tissue. Techniques such as intensity-modulated radiation therapy (IMRT), image-guided radiation therapy (IGRT), stereotactic radiosurgery (SRS), and stereotactic body radiation therapy (SBRT or stereotactic ablative radiotherapy - SABR) have transformed the delivery of radiotherapy, and the trend is the increasing use of hypofractionated schedules. With stereotactic techniques, single doses as high as 20 to 24 Gy or highly hypofractionated schemes such as 54 to 60 Gy in three fractions can be safely delivered in small targets.

Published papers have suggested that SBRT regimens can promote an immune response, mediating anti-tumorigenic effects 60. The generation of an effect in the TME that can initiate an immunological response may support this theory 61. Two aspects will be important in the elucidation of this effect are evaluation of immune cell response to high-dose SBRT regimens and cancer stem cell clearance to exclude any effect of endothelial damage 62.

Cancer stem cells have been implicated in the radioresistant characteristics of tumors. This cell type may be responsible for tumor recurrence following fractionated radiotherapy 62,63. Cancer stem cells increase the activation of the AKT/mTOR pathway, which regulates cell proliferation and survival 62. Therefore, the efficiency of SBRT with a high dose can be responsible for the increase in the number of cell targets damaged and for the ablation of cancer stem cells, which is a focus of radioresistance 61,62.

Changes in the TME could also have a marked impact on the response of tumor cells to treatment 63,64. Not only may the effects on endothelial cells and tumor vasculature contribute to tumor growth or control, but other stroma cell populations may also be involved. The mechanisms responsible for the radiation effect and tumor relapse following high-dose radiotherapy have been explored in more recent experiments 64,65.

### Combination of particle radiotherapy with immunotherapy

Photon-based radiation (i.e., gamma rays and X-rays) and small particles, such as electron beams, have been used to treat all types of cancer and some nonmalignant disorders. Heavy particle radiotherapy (i.e., protons and carbon ions) has specific physical properties and biological effectiveness that are very attractive in the clinic and are now becoming a more popular treatment modality 65.

Particle beams may be as effective as, or more advantageous than, photon beams in immune combination therapy. The same relative biological effective (RBE) dose induces different biological responses when particles are used compared to photon beams (3 times more efficient). Another interesting point observed in several studies was the effect of particle beams on the tumor metastatic potential. In some cases, treatment with X-rays increased the metastatic potential of a tumor, while carbon ion beams effectively suppressed it 66.

Sublethal doses delivered with carbon ion beams inhibited *in vitro* angiogenesis 67 and the expression of angiogenesis mediators 68,69. In immune-competent mouse models, lung metastasis was significantly suppressed by carbon ion irradiation 66,70.

When heavy particles are used compared to conventional photons, there is an inverted depth-dose profile, with a low entrance dose, followed by a peak dose at a certain depth (Bragg peak), with a sharp fall-off afterwards that allows for a more precise dose delivery 66,71. Recent studies also suggest another advantage of both protons and carbon ions because they have unique biological properties when compared with photons 66,71,72. They have the characteristic of high-linear energy transfer (LET) of these particles 66,72,73. Considering these special characteristics (physical and biological), particle therapy could have important and different effects on immune alterations. The response caused would define the type and magnitude of local and systemic (abscopal) immune effects 74.

A few preclinical *in vitro* and *in vivo* studies investigated the effects of particle radiotherapy on the modification of tumor cell phenotypes and whether this would increase their sensitivity to immune surveillance. Proton irradiation modulates several processes critical in tumor growth and progression, including angiogenesis and immunogenicity. Decreased levels of factors that may influence the tumor response to radiation and the host immune response, such as vascular endothelial growth factor (VEGF), IL-6, IL-8, and hypoxia-inducible factor 1-alpha (HIF 1-α), were observed in lung carcinoma cells exposed to proton irradiation 74,75. Likewise, sublethal doses of proton or photon irradiation induced a similar increase in the levels of surface molecules involved in T-cell recognition as well as the translocation of calreticulin to the tumor cell surface 76. These changes in calreticulin are critical for increased sensitivity to T cell killing and render tumor cells more sensitive to T cell-mediated cell death. In a human *in vitro* model, the levels of HMGB1 in different human cancer cell lines were increased by carbon ion radiation. However, when the dose was changed to the levels used in the clinic, this effect was comparable to that produced by irradiation with X-rays 77.

Carbon ion exposure also induces antitumor immunity and abscopal effects in some cases, as described in preclinical *in vivo* studies. When compared with photon irradiation, the number of distant lung metastases was reduced, and higher expression levels of membrane-associated immunogenic molecules were observed in carcinoma models in immunocompetent mice 78. In both studies, immunocompetent mice received bone marrow DCs and irradiation. In addition to the potential of carbon ion exposure alone to activate DCs, the combined treatment showed a synergistic effect. However, the combination of DC immunotherapy with photon radiation was not able to induce the same effects. These studies suggest that carbon ion therapy with the same dose might generate a stronger activation of the immune system than conventional photon radiotherapy 79,80.

### Perspectives in treatment with new technologies

In recent years, nanotechnology has emerged as a new scientific point of interest. It is another tool in medical treatments that is emerging as a promising therapeutic plane. There are significant numbers of clinical benefits reported. The nanomaterials present wide potential for medical use, including tissue engineering protein detection and drug and/or gene delivery. The inclusion of nanoparticles (NPs) into both diagnostic and radiation therapy settings, emphasizing their use as potential agents in the combination of diagnosis and therapeutics, may be promising 81,82.

### Nanotechnology and radiation therapy

Nanotechnology may be incorporated into ionizing radiation treatments 82. Radiosensitizing NPs, for example, have the potential to increase the radiation dose delivered to tumor cells while sparing adjacent normal tissue, optimizing the therapeutic ratio in *in vivo* studies 83. Elements with a high atomic number (Z) have a strong photoelectric absorption when irradiated with X-rays. High-Z NPs (particularly gold NPs) were initially considered radiation contrast agents. In addition, a potential benefit of the combination of these NPs with low energy (kV) X-rays was observed. The association produces a radiation dose increase, with a potential increase in cell death. Likewise, successful radiosensitization was observed in early *in vivo* work related to this issue 84. However, it was also observed that the increase in the physical dose does not fully explain the observed large increase in radiosensitization 85.

These findings demonstrate that the factors that impact NP radiosensitization still need to be better understood 82. It seems that, together with their impact on the physical dose distribution, NPs also induce some degree of biological sensitization of tumor cells. Physically, high-Z NPs promote an increase in total dose absorption and the release of a large number of lower energy X-rays and Auger electrons due to the photoelectric effect. The energy released by these particles is deposited very nearby, resulting in highly localized damage. This is responsible for an increased biologic effect and results in higher damage 82. Some NP preparations may also produce additional biological effects, such as mitochondrial stress or the production of reactive oxygen species, that may also enhance the effectiveness of radiation 82.

The development of the targeted uptake of NPs may help to increase the dose to the tumor without changing the dose to the adjacent normal tissue. This effect is relevant regarding SBRT treatments, in which the highly hypofractionated treatment schedule requires a high degree of precision to avoid any geometric mistakes, with impacts on tumor control and toxicity. Therefore, the combination of NPs with SBRT not only has the biological potential of increasing ICD but also has improved the therapeutic ratio 82,83.

Nanoparticles have also been explored as vectors for radiation protection agents in radiotherapy 83,86. Reduction of bone marrow toxicity during external beam radiotherapy was observed in mice after the intravenous administration of melanin-coated NPs, with an influence on tumor control 87.

### Clinical implications / on-going trials

The potential benefits of the combination of radiotherapy with drugs that cause immune system activation was already described in the 1970s 87. However, only in the past 10 years has this type of treatment started to be developed. Currently, there is still a lack of information about the ideal combination of ionizing radiation with immunotherapy, and there are no recommended “off-protocol” approaches already established for routine patient management.

The main objective of the current ongoing trials is to evaluate the abscopal effect of the combination of ionizing radiation with immunotherapy, mainly in patients with advanced disease.

Different vaccines and immune checkpoint inhibitors are combined with radiotherapy that is delivered to the primary tumor or metastatic sites in oligometastatic disease. Among these, vaccinia prostate-specific antigen (rV-PSA), Toll-like receptor (TLR)-7 agonist, cytokine FLT3L ligand (CDX-301), anti-programmed cell death-1 (PD-1), anti-programmed cell death ligand-1 (PD-L1), and anti-cytotoxic T-lymphocyte associated protein 4 (CTLA-4) antibodies, IL-2, recombined human granulocyte-macrophage colony stimulating factor (rhGM-CSF), and tyrosine kinase inhibitors (TKIs) have been identified and tested 44,88-92.

The trials involve distinct tumor sites, in different stages of the disease, most frequently in the metastatic setting. All are phase 1 or 2, and radiotherapy is preferentially delivered with SBRT.

Some of these ongoing trials from New York University (NYU), the Earle A. Chiles Research Institute (EACRI), the Providence Cancer Center (Providence Portland Medical Center, Oregon) (PH&S IRB), Stanford University, the National Institute of Health/National Cancer Institute (NIH/NCI) and Thomas Jefferson University were already well described in a recent review 93. Table 1 displays other ongoing clinical trials registered at “clinicaltrials.gov” that are studying the abscopal effect of ionizing radiation combined with immunotherapy. A glossary of the drugs used in these trials is available in the Appendix.

Over time, patients enrolled in these trials are carefully monitored regarding their immune function. We hope that important data about the pro-immunogenic effects of the combination of different immune modulators with radiation in different disease settings will soon be available.

## FINAL REMARKS

Radiotherapy technology is progressively evolving to more precise and safe treatments, with better normal tissue sparing and the potential to deliver high ablative doses to the tumor.

Molecular biology studies are allowing the development of many anti-cancer agents with more specific targeted treatments.

The effects of these treatments in the immune system and the benefits of these effects are still under intensive research. Paradoxical effects of stimulation or immunosuppression are observed in different scenarios and may contribute to the success or lack of success of the different treatment approaches.

Interaction of novel radiation therapy technologies with the newly developed targeted agents are promising strategies for cancer treatment. Hypofractionated radiotherapy seems to be more effective regarding immunotherapy. Nevertheless, better drug–radiation combinations, timing, sequences, radiation doses and fractionation have yet to be defined.

## AUTHOR CONTRIBUTIONS

Carvalho HA and Villar RC contributed equally to the bibliographic review and manuscript writing.

## Figures and Tables

**Table 1:** Currently open clinical trials of immunotherapy and radiation (available at www.clinicaltrials.gov, Dec 2017).

ID / Study title	Tumor site / stage	Treatment combination	Country
NCT03113851 Abscopal Effect of Radiation in Combination With rhGM-CSF for Metastatic Non-small Cell Lung Cancer	Lung cancer metastatic	RT 35Gy / 10 fractions	China
		IT rhGM-CSF	
NCT03323424 Phase II Trial Assessing the Efficacy of Immuno-Radiation Abscopal Effect in Patients With Metastatic Cancers (IRAM)	Breast, colorectal, or upper aerodigestive tract metastatic	SBRT for metastasis: 45Gy / 3 fractions (hepatic and pulmonary) 27Gy / 3 fractions (bone) 33Gy / 3 fractions (intra-cranial)	France
		IT Therapies capable of ADCC	
NCT02542137 Abscopal Effect for Metastatic Small Cell Lung Cancer	Small cell lung cancer	RT 35Gy / 10 fractions	China
		IT Thymosin-α-1	
NCT02535988 Abscopal Effect for Metastatic Colorectal Cancer	Metastatic colorectal cancer	RT 35Gy / 10 fractions	China
		IT Thymosin-α-1	
NCT02542930 Abscopal Effect for Metastatic Non-small Cell Lung Cancer	Non-small cell lung cancer	RT 35Gy / 10 fractions	China
		IT Thymosin-α-1	
NCT03354962 Induction of Immune-mediated aBscOpal Effect thrOugh STEreotactic Radiation Therapy in Metastatic Melanoma Patients Treated by PD-1 + CTLA-4 Inhibitors	Melanoma	RT SBRT (recommended optimal dose)	France
		IT Anti PD-1 + anti-CTLA-4	
NCT02334709 Phase I/II Trial of Stereotactic Body Radiotherapy With Concurrent Fixed Dose Tyrosine Kinase Inhibitors in Metastatic Renal Cell Carcinoma: Dose Limiting Toxicity and Abscopal Effect.	Renal cell carcinoma	SBRT (3 dose levels): 24Gy / 3 fractions 30Gy / 3 fractions 36Gy / 3 fractions	Belgium
		IT TKIs	
NCT02623595 A Study of SBRT in Combination With rhGM-CSF for Stage IV NSCLC Patients Who Failed in Second-line Chemotherapy	Non-small cell lung cancer	RT SBRT 50Gy / 5 fractions	China
		IT rhGM-CSF	
NCT02406183 Trial of SBRT With Concurrent Ipilimumab in Metastatic Melanoma	Melanoma	RT SBRT (3 dose levels): 24Gy / 3 fractions 30Gy / 3 fractions 36Gy / 3 fractions	Belgium
		IT anti-CTLA-4	
NCT02830594 Pembrolizumab and Palliative Radiation Therapy in Treating Patients With Metastatic Esophagus, Stomach, or Gastroesophageal Junction Cancer	Metastatic esophageal, stomach, or gastroesophageal junction cancer	RT SBRT (3 dose levels): 24Gy / 3 fractions 30Gy / 3 fractions 36Gy / 3 fractions	United States
		IT Anti-PD-1	
NCT02562625 Trial of Pembrolizumab and Radiotherapy in Melanoma (PERM)	Melanoma	RT 24Gy / 3 fractions	England
		IT Anti-PD-1	
NCT02538471 LY2157299 Monohydrate (LY2157299) and Radiotherapy in Metastatic Breast Cancer	Metastatic breast cancer	RT 22.5Gy / 3 fractions	United States
		IT TGF-β receptor type-1 kinase inhibitor	
NCT02976740 SBRT Combination With rhGM-CSF and Tα1 for Stage IV NSCLC Patients Who Failed in Second-line Chemotherapy	Metastatic lung cancer	RT SBRT 50Gy / 4-10 fractions	China
		IT rhGM-CSF	
NCT02115139 GEM STUDY: Radiation And Yervoy in Patients With Melanoma and Brain Metastases (GRAY-B)	Melanoma	RT 30Gy / 10 fractions (whole brain)	Spain
		IT anti-CTLA-4	

Abbreviations: RT, radiotherapy; IT, immunotherapy; SBRT, stereotactic body radiotherapy; ADCC, antibody-dependent cell cytotoxicity; rhGM-CSF, recombinant human granulocyte-macrophage colony stimulating factor; TGF-β, tumor growth factor-beta; anti-CTLA-4, cytotoxic T-lymphocyte associated protein 4; anti-PD-1, anti-programmed cell death-1.
